# Premature ventricular complexes increase with heart rate in patients with mitral valve prolapse^☆^

**DOI:** 10.1016/j.ijcha.2026.101925

**Published:** 2026-04-15

**Authors:** Cecilie Bugge, Christian K. Five, Tariq Ahmed, Petter M.J. Skar, Anna Isotta Castrini, Margareth Ribe, Nina E. Hasselberg, Lars A. Dejgaard, Mathis K. Stokke, Kristina H. Haugaa, Eivind W Aabel

**Affiliations:** a ProCardio Center for Innovation, Department of Cardiology, Oslo University Hospital, Rikshospitalet, PO Box 4950 Nydalen, 0424 Oslo, Norway; b Institute of Clinical Medicine, Faculty of Medicine, University of Oslo, PO Box 1171 Blindern, 0318 Oslo, Norway; c Institute for Experimental Medical Research, Oslo University Hospital and University of Oslo, PB 4956 Nydalen, 0424 Oslo, Norway

**Keywords:** Mitral valve prolapse, Ventricular arrhythmia, Premature ventricular complexes, Holter monitoring, Cardiomyopathy, Mitral annular disjunction

## Abstract

**Background:**

Patients with mitral valve prolapse (MVP) are at risk of ventricular arrhythmias (VAs), ranging from premature ventricular complexes (PVCs) to life-threatening VAs. The association between PVC burden and heart rate in patients with MVP is not known. We aimed to identify the association between PVC burden and heart rate in patients with MVP.

**Methods:**

In this this cross-sectional ambispective case control study we included MVP patients with available Holter monitorings. We defined PVC profiles as fast-heart-rate-dependent-PVC (F-HR-PVC) in case of positive correlation with heart rate, slow-heart-rate-dependent-PVC (S-HR-PVC) in case of negative correlation, and independent-heart-rate-PVC (I-HR-PVC) when no correlation was found. For comparison, we included a control group of age- and sex-matched patients with idiopathic PVCs.

**Results:**

We included 70 patients with MVP (48 years [interquartile range 35–58], 79% female) and 70 age- and sex-matched patients with idiopathic PVCs. A total of 153 Holter monitorings from patients with MVP were analysed and compared to 70 Holter monitorings from patients with idiopathic PVCs. In the MVP group, we found F-HR-PVC in 44 (63%) patients, I-HR-PVC in 24 (34%) and S-HR-PVC in 2 (3%). MVP patients had more frequently F-HR-PVC and less frequently S-HR-PVC than the control group (p < 0.05 for both). MVP patients with F-HR-PVC had higher rate of NSVTs (incidence rate ratio 2.9 [95% confidence interval 1.1–7.8], p = 0.03) compared to I-HR-PVC.

**Conclusion:**

Fast-heart-rate-dependent-PVC was the most common PVC profile in MVP patients, and slow-heart-rate-dependent-PVC was rare. These findings suggest a catecholamine-sensitive mechanism acting as trigger for ventricular arrhythmias in MVP patients.

## Highlights

• Most patients with MVP had PVCs that positively correlated with increasing heart rate.
• This suggests a catecholamine-sensitive mechanism acting as trigger for ventricular arrhythmias in these patients.
• PVCs associated with decreasing heart rate was rare.
• Patients with PVCs associated with increasing heart rate did not have higher risk of severe ventricular arrhythmia.
• Patients with PVCs associated with increasing heart rate had a higher burden of non-sustained ventricular tachycardias.

## Graphical abstract

We included 70 patients with mitral valve prolapse and 70 age and sex-matched patients with idiopathic PVCs and ≥100 PVCs per 24-hours on Holter monitoring. In MVP patients, Fast-HR-dependent-PVC was the most common PVC profile. HR = heart rate, IQR = interquartile range, MVP = mitral valve prolapse, NSVT = non-sustained ventricular tachycardia, PVC = premature ventricular complexes, VA = ventricular arrhythmia.

**Figure:**
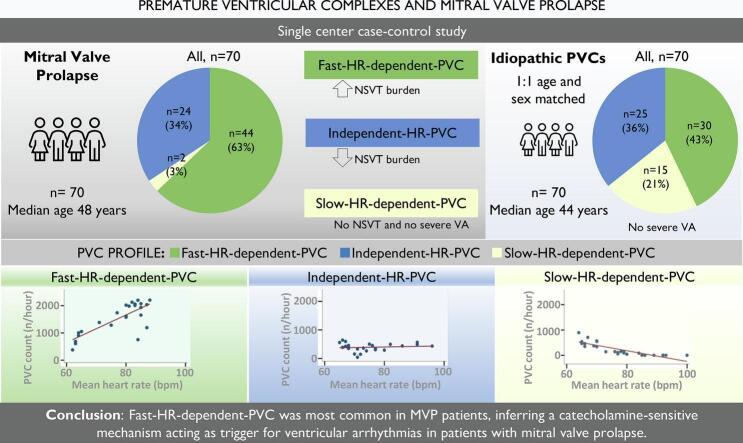

## 1 Introduction

A subset of patients with mitral valve prolapse (MVP) have recurrent ventricular arrhythmias, ranging from frequent PVCs to aborted cardiac arrest. This phenotypic expression is termed arrhythmic mitral valve prolapse (AMVP) [1], and is seen as a significant potential cause of unexplained sudden cardiac death [2], [3], [4], [5]. Risk stratification in AMVP is a major challenge, although several risk factors have been identified, such as bileaflet MVP, mitral annulus disjunction, severe mitral regurgitation, dilated left ventricle, and myocardial fibrosis in the papillary muscles or the inferobasal left ventricle [1]. In addition, a high burden of premature ventricular complexes (PVCs), and a PVC burden ≥ 5% is associated with increased mortality and subsequent ventricular tachyarrhythmias in patients with AMVP [1], [6], [7].

Correlations between heart rate and PVC burden was first described in 1982 [8]. In treatment of idiopathic PVCs betablockers are preferred in case of a higher burden of PVCs with higher heart rate [9]. However, the association between PVC burden and heart rate in patients with MVP is not known. It remains uncertain whether the relationship between PVC burden and heart rate can be utilized to estimate the risk of subsequent ventricular tachyarrhythmias.

In the present study, we aimed to explore the relationship between PVC burden and heart rate in patients with MVP and compare this relationship to patients with idiopathic PVCs. We also wanted to establish whether different PVC profiles were associated with severe ventricular arrhythmias in patients with MVP. We hypothesized that increasing PVC burden with increasing heart rate was less frequently found in patients with MVP than in patients with idiopathic PVCs, and that this PVC profile would be associated with increased risk of ventricular tachyarrhythmias.

## 2 Methods

### 2.1 Study population and recruitment

In this cross-sectional ambispective case control study, we included patients with MVP. Patients were recruited from two centres (Oslo University Hospital and Drammen Hospital) from 2014 to 2023. All eligible patients with any degree of MVP and/or mitral annular disjunction, irrespective of the grade of mitral regurgitation were evaluated at Oslo University Hospital and included in an MVP/mitral annular disjunction cohort. Evaluations performed at inclusion in this cohort has been described previously [7], [10], [11], [12]. In short, patients were evaluated with clinical examination, 12-lead electrocardiogram (ECG), 24-hour Holter monitoring, exercise stress ECG, transthoracic echocardiography, and cardiac magnetic resonance imaging [10]. Antiarrhythmic drug therapy was not discontinued before Holter monitoring for the MVP/mitral annular disjunction patients. We excluded patients with previous mitral valve surgery, as well as other known aetiologies of arrhythmia and performed genetic testing to exclude channelopathies and cardiomyopathies in patients with severe ventricular arrhythmia, on clinical indication. In this sub-study, we included all patients with confirmed MVP and at least one Holter monitoring exceeding 100 PVCs per 24-hours.

We included a 1:1 age and sex matched control group consisting of patients with idiopathic PVCs evaluated for catheter ablation at Oslo University Hospital from 2020 to 2024. The patients were included based on presence of PVCs with inferior axis and left bundle branch block pattern, as well as no sign of underlying heart disease from 12-lead ECG, 24-hour Holter monitoring, exercise stress ECG, transthoracic echocardiography and cardiac magnetic resonance imaging. Antiarrhythmic drug therapy was discontinued one week before Holter monitoring for the patients with idiopathic PVCs. Patients with MVP as an incidental finding in the control group were excluded, as were patients with repetitive NSVT or a history of more severe ventricular arrhythmias.

The study complied with the Declaration of Helsinki and was approved by the Regional Committee for Medical Research Ethics (2015/596/REK Nord and 2020/153850/REK Sør-Øst A). All study participants provided written informed consent.

### 2.2 Holter monitoring and classification of PVC profiles

**Fig. 1:**
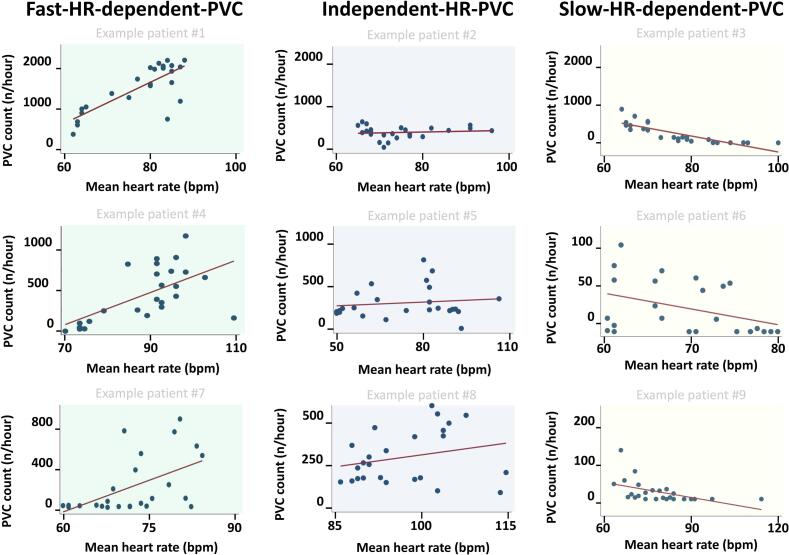
Examples of PVC profiles on Holter monitoring. Examples of 9 patients with different PVC profiles. Each panel represent one Holter monitoring for an example patient with the PVC profile. The panels show the distribution between PVC count/hour and the mean heart rate/hour with fitted line. HR = heart rate, PVC = premature ventricular complexes.

Raw data from the 24-hour Holter monitoring was manually extracted using Medilog® DARWIN2 (Schiller, Baar, Switzerland). We analysed all available Holter monitorings with ≥100 PVCs. We categorized patients into three profiles based on the relationship between hourly PVC burden and the hourly mean heart rate. We defined the PVC profiles as (1) fast-heart-rate-dependent-PVC (F-HR-PVC) when there was a statistically significant (p < 0.05) positive correlation, (2) slow-heart-rate-dependent-PVC (S-HR-PVC) when there was a significant negative correlation, and (3) independent-heart-rate-PVC (I-HR-PVC) when no correlation between PVC and heart rate was found (Fig. 1) [8], [13], [14], [15].

The PVC burden was defined as the percentage PVCs of the total heart beats per 24-hours. The NSVT burden was defined as the number of NSVTs per 24-hours, reported in the patients with NSVT present. In case of repeated Holter monitorings, a mean/median for PVC burden, NSVT burden, minimum, mean and maximum heart rate for the individual patient was generated. We collected data on antiarrhythmic drug therapy.

### 2.3 Ventricular arrhythmia

Non-sustained ventricular tachycardia (NSVT) was defined as ≥3 consecutive ventricular beats with heart rate >110 beats/min lasting <30 s, and NSVT burden as the total number of NSVT episodes registered per 24 h. Sustained ventricular tachycardia was defined as consecutive ventricular beats with heart rate >110 beats/min lasting ≥30 s. AMVP was defined according to European Heart Rhythm Association expert consensus (MVP and either ≥5% PVCs or the presence of NSVT, sustained ventricular tachycardia or ventricular fibrillation) [1].

We defined severe ventricular arrhythmia as aborted cardiac arrest, appropriate shock by an implantable cardioverter defibrillator, sustained VT or NSVT with haemodynamic instability. Severe ventricular arrhythmias were recorded from medical history, implantable cardioverter-defibrillator interrogation, monitoring with implantable loop recorder or clinical follow-up.

PVC morphology from 12-lead ECG and 12-lead stress ECG was categorized in left or right bundle branch block morphology with superior or inferior frontal axis [16]. Outflow tract PVCs were defined as having left bundle branch block-like morphology with inferior frontal axis, and left ventricular PVCs as having right bundle branch block-like pattern with either superior or inferior frontal axis. T-wave inversion was defined as present if seen in ≥2 adjacent ECG leads. T-wave inversion in the inferior wall was defined as T-wave inversion in lead II, III and/or aVF.

### 2.4 Echocardiography and cardiac magnetic resonance imaging

Cardiac volumes and function were measured according to guidelines [17], [18], [19], and valvular pathology was graded using echocardiography [19]. We defined MVP as superior displacement ≥2 mm of any part of the mitral leaflet beyond the mitral annulus on echocardiography in the parasternal long-axis view [1]. Inferolateral mitral annular disjunction was defined as ≥1 mm disjunction measured between the left atrial wall at the junction of the posterior mitral leaflet and the top of the left ventricular myocardium, using both echocardiography and cardiac magnetic resonance imaging [10]. Late gadolinium enhancement was reported if present [10].

### 2.5 Statistical analysis

Values were expressed as mean ± standard deviation (SD), frequencies (%), or median with interquartile range (IQR) as appropriate. Groups were compared with independent Student’s *t*-test, Mann–Whitney *U* test, χ^2^, Fisher exact tests and one-way ANOVA, as appropriate. Statistical comparisons were made only between the F-HR-PVC and the I-HR-PVC profile. Correlation between hourly mean heart rate and PVC burden was addressed by univariable mixed linear regression with random effects on individual Holter monitoring. To assess the correlation between PVC profile and NSVT burden, we used negative binominal regression with random effects on individual level. Two-sided P-values < 0.05 were considered significant (Stata/SE v18.0, StataCorp LLC, TX, USA).

## 3 Results

### 3.1 Study population, patient characteristics and risk factors

**Fig. 2:**
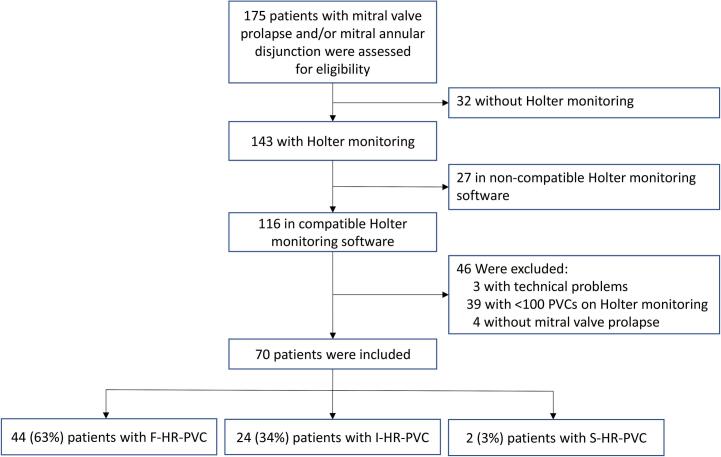
Study flow chart. In this study, we included 70 patients from our cohort of MVP/mitral annular disjunction patients that had verified MVP and available Holter monitoring with ≥100 PVCs per 24-hours. F-HR-PVC = Fast-heart-rate-dependent-PVC, I-HR-PVC = Independent-heart-rate-PVC, PVCs = premature ventricular complexes, S-HR-PVC = Slow-heart-rate-dependent-PVC.

**Fig. 3:**
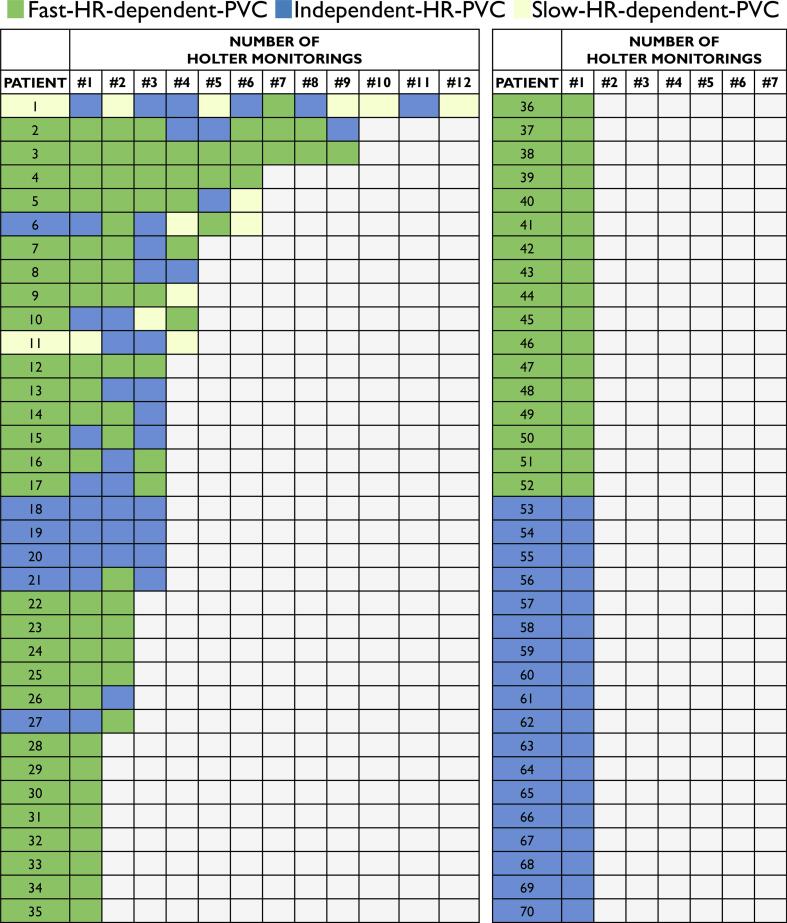
PVC profiles in 70 MVP patients. Each line represents one individual patient with MVP, with corresponding number of Holter monitoring and the PVC profile on that Holter monitoring. F-HR-PVC = Fast-heart-rate-dependent-PVC, I-HR-PVC = Independent-heart-rate-PVC, MVP = mitral valve prolapse, PVC = premature ventricular complexes, PVCs = premature ventricular complexes, S-HR-PVC = Slow-heart-rate-dependent-PVC.

We included 70 patients with MVP (median age 48 years [IQR 35–58], 79% female) (Table 1, Fig. 2), of which 55 (79%) fulfilled the European Heart Rhythm Association diagnostic criteria for AMVP [1] (22 [31%] due to PVC ≥ 5%, 54 [77%] due to NSVT, ventricular tachycardia or ventricular fibrillation and 21 [30%] fulfilled both criteria). A total of 153 Holter monitorings was analysed, where 27 patients had repeated Holter monitorings (minimum 1 Holter monitoring, maximum 12 Holter monitorings) (Fig. 3). The median PVC burden was 1.2% per 24-hours (IQR 0.4–3.6).

**Table 1:** Clinical characteristics of 70 MVP patients and 70 idiopathic PVC patients.

	MVP (n = 70)	Idiopathic PVCs (n = 70)
Age at inclusion, years (IQR)	48 (35–58)	44 (34–56)
Female, n (%)	55 (79)	53 (76)
Race		
Caucasian, n (%)	70 (100)	70 (100)
BMI, kg/m^2^ (IQR)	23 (21–25)	26 (22–28)
Medical history		
Diabetes, n (%)	0 (0)	1 (1)
Hypertension, n (%)	4 (6)	8 (11)
Coronary artery disease, n (%)	1 (1)	0 (0)
Atrial fibrillation, n (%)	10 (14)	4 (6)
Valvular heart disease		
No mitral regurgitation, n (%)	13 (18)	29 (41)
Mild mitral regurgitation, n (%)	37 (53)	40 (57)
Moderate mitral regurgitation, n (%)	14 (20)	1 (1)
Severe mitral regurgitation, n (%)	6 (9)	0 (0)
Severe aortic stenosis, n (%)	0 (0)	0 (0)
Severe aortic regurgitation, n (%)	0 (0)	0 (0)
Left ventricular ejection fraction, %	57 ± 6	56 ± 5
<50%, n (%)	6 (9)	4 (6)
Severe ventricular arrhythmia, n (%)	13 (19)	0 (0)
Antiarrhythmic drug therapy**		
None, n (%)	22 (31)	27 (39)
Betablocker, n (%)	42 (60)	33 (47)
Flecainide, n (%)	8 (11)	15 (21)
Calcium channel blocker, n (%)	1 (1)	5 (7)
Digoxin, n (%)	2 (3)	0 (0)

**Holter monitoring**		
PVC profile		
F-HR-PVC, n (%)	44 (63)	30 (43)
I-HR-PVC, n (%)	24 (34)	25 (35)
S-HR-PVC, n (%)	2 (3)	15 (21)
PVCs, n per 24-h (IQR)	1185 (291–3914) *	9026 (2797–16682)
PVC burden, % per 24-h (IQR)	1.2 (0.4–3.6) *	8.0 (3.0–16.2)
Right bundle branch block, n (%)	45 (64)	0 (0)
Superior axis, n (%)	38 (54)	0 (0)
Inferior axis, n (%)	19 (27)	0 (0)
Left bundle branch block inferior axis, n (%)	31 (44)	70 (100)

Values are presented as n (%) or median (IQR).

* The PVC burden in the MVP group is based on repeated Holter monitoring, while the idiopathic PVC group is based on a single monitoring.

** Patients could receive treatment with more than one antiarrhythmic drug.

BMI = Body mass index, F-HR-PVC = Fast-heart-rate-dependent-PVC, I-HR-PVC = Independent-heart-rate-PVC, IQR = interquartile range, MVP = mitral valve prolapse, PVC = premature ventricular complexes, S-HR-PVC = Slow-heart-rate-dependent-PVC, VT = ventricular tachycardia.

### 3.2 PVC profiles on Holter monitoring

**Fig. 4:**
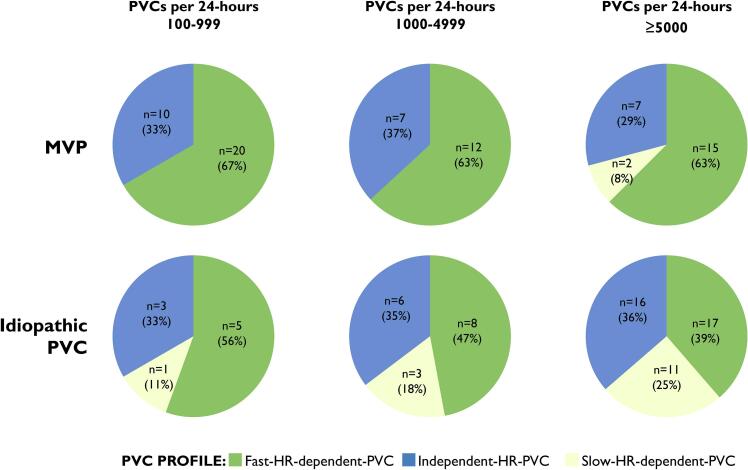
Distribution of PVC profiles based on PVC burden. We found similar distribution of PVC profiles in a subanalysis looking at patients with different PVC burden. HR = heart rate, PVC = premature ventricular complexes.

We found F-HR-PVC in 44 patients (63%), I-HR-PVC in 24 patients (34%) and S-HR-PVC in 2 (3%) in patients with MVP (Graphical Abstract, Table 2, Fig. 1, Fig. 2, Fig. 3, Fig. 4). The maximum heart rate was higher in the F-HR-PVC profile compared to the I-HR-PVC profile (17 beats per minute [bpm] higher [95% CI 7–29], p = 0.001), which was also significant when adjusted for NSVT burden (17 bpm higher [95% CI 6–28], p = 0.002). F-HR-PVC were also associated with lower minimum heart rate (5 bpm lower [95% CI − 9.5 to −0.5], p = 0.03) compared to I-HR-PVC.

**Table 2:** Clinical characteristics by different PVC profiles in MVP patients.

	**All** **(n = 70)**	**F-HR-PVC** **(n = 44)**	**I-HR-PVC** **(n = 24)**	**S-HR-PVC** **(n = 2)**	P-value *
Number of Holter monitorings per patient, mean (min–max)	2 (1–12)	1 (1–9)	2 (1–6)	8 (4–12)	
Age at inclusion, years (IQR)	48 (35–58)	47 (35–59)	52 (35–59)	32 (29–35)	0.67
Female, n (%)	55 (79)	35 (80)	19 (79)	1 (50)	0.97
EHRA AMVP diagnosis, n (%)	55 (79)	32 (72)	21 (89)	2 (100)	0.23
Severe ventricular arrhythmia (%)	13 (19)	8 (18)	5 (21)	0 (0)	0.79
T-wave inversions inferior wall, n (%)	10 (14)	5 (11)	4 (17)	1 (50)	0.71

**Echocardiography**					
Left ventricular ejection fraction (%)	57 ± 6	57 ± 6	56 ± 6	59 ± 2	0.37
Mitral regurgitation, (grade)					0.77
None, n (%)	13 (19)	6 (14)	6 (25)	1 (50)	
Mild, n (%)	37 (53)	23 (52)	13 (54)	1 (50)	
Moderate, n (%)	14 (20)	9 (20)	5 (21)	0 (0)	
Severe, n (%)	6 (9)	6 (14)	0 (0)	0 (0)	
Inferolateral mitral annular disjunction, n (%)	62 (88)	39 (89)	21 (89)	2 (100)	1.00
Inferolateral mitral annular disjunction, length in mm (IQR)	6 (4–8)	6 (4–8)	5 (3–8)	6 (6–6)	0.52
Bileaflet MVP, n (%)	40 (57)	26 (59)	14 (58)	0 (0)	0.95

**Cardiac magnetic resonance imaging (n = 47)**					
Late gadolinium enhancement, n (%)	17 (37)	9 (33)	7 (41)	1 (50)	0.60
Papillary muscles, n (%)	11 (24)	5 (19)	5 (29)	1 (50)	0.32

**PVC morphology (n = 69)**					
Right bundle branch block, n (%)	45 (54)	30 (70)	14 (58)	1 (50)	0.35
Superior axis, n (%)	38 (58)	25 (60)	12 (55)	1 (50)	0.70
Inferior axis, n (%)	19 (29)	11 (26)	8 (36)	0 (0)	0.40
Left bundle branch block inferior axis, n (%)	31 (45)	21 (49)	8 (33)	2 (100)	0.22

**Holter monitoring**					
PVCs**, n per 24-h (IQR)	1185 (291–3914)	948 (274–4097)	1917 (415–4125)	1777 (1754–1799)	0.39
PVC burden**, % per 24-h (IQR)	1.2 (0.4–3.6)	1.0 (0.3–3.8)	1.7 (0.4–4.1)	1.7 (1.6–1.8)	0.38
NSVT present**, n (%)	38 (53)	22 (50)	14 (58)	1 (50)	0.77
NSVT burden**, n (IQR)	2 (1–14)	2 (1–11)	2 (1–4)	76 (75–76)	0.62
3 complexes, n (IQR)	3 (1–14)	2 (1–10)	2 (1–4)	53 (33–73)	0.71
≥4 complexes, n (IQR)	1 (1–9)	1 (1–1)	5 (1–21)	43	0.64

Values are presented as n (%), median (IQR) or mean ± SD. The P-values were calculated by means of Student *t*-test, one-way ANOVA, Mann–Whitney *U* test, χ^2^ test or Fisher exact test as appropriate.

* P-values are comparisons between F-HR-PVC and I-HR-PVC.

** The reported PVC burden and NSVT burden is the mean reported based on single Holter monitoring or repeated Holter monitorings if available.

AMVP = arrhythmic mitral valve prolapse, EHRA = European Heart Rhythm Association, F-HR-PVC = Fast-heart-rate-dependent-PVC, I-HR-PVC = Independent-heart-rate-PVC, IQR = interquartile range, MVP = mitral valve prolapse, NSVT = non-sustained ventricular tachycardia, PVC = premature ventricular complexes, S-HR-PVC = Slow-heart-rate-dependent-PVC, VT = ventricular tachycardia.

PVCs with right bundle branch morphology, suggesting an origo in the left ventricle, was found in 45 (54%) patients, and with left bundle branch block morphology with inferior axis, suggesting an origo from the outflow tract, in 31 (45%) patients, with similar prevalence of different PVC morphologies in the F-HR-PVC and I-HR-PVC profiles. Only one patient had a sustained ventricular tachycardia on the 24-hour Holter monitoring and had a F-HR-PVC profile.

Mitral valve surgery was performed in one patient during follow-up (patient #5 in Fig. 3). Three Holter monitorings were recorded before surgery, all with a F-HR-PVC profile. After surgery, an additional 3 Holter monitorings were recorded, 1 with a F-HR-PVC profile, 1 with a I-HR-PVC profile, and 1 with a S-HR-PVC profile.

### 3.3 Ventricular arrhythmia and PVC profile

Patients with F-HR-PVC had higher rate of NSVTs compared to I-HR-PVC (incidence rate ratio [IRR] 2.9, 95% confidence interval [CI] 1.1–7.8, p = 0.03). However, the PVC burden was similar in the F-HR-PVC profile and the I-HR-PVC profile (1.0% per 24-hours [IQR 0.3–3.8] vs 1.7% per 24-hours [IQR 0.4–4.1], p = 0.38). Six (10%) patients had NSVT on exercise stress ECG, and all patients had a F-HR-PVC profile.

Severe ventricular arrhythmia had occurred in 13 (19%) patients with MVP, of which 8 (11%) had aborted cardiac arrest, 2 (3%) had sustained VT, 1 (1%) had ICD shock from primary preventive ICD and 2 (3%) had NSVT with hemodynamic instability (Supplementary Table 1). We found no association between different PVC profiles and the presence of severe ventricular arrhythmia (F-HR-PVC 63% vs 62%, I-HR-PVC 33% vs 38%, S-HR-PVC 4% vs 0%, p > 0.05 for all profiles) (Table 2).

Of the 13 patients with severe ventricular arrhythmia, 12 patients (92%) could account for the circumstance of the event. One had an aborted cardiac arrest during exercise, one had NSVT with hemodynamic instability during exercise, and the other 10 had events during wakeful rest. Patients with severe ventricular arrhythmia had a higher PVC burden (log coefficient 1.40, 95% CI 0.66–2.15, p < 0.001) and a higher rate of NSVTs (IRR 10.2, 95% CI 2.1–50.2, p = 0.004) compared to those without severe ventricular arrhythmia.

Among the 55 MVP patients fulfilling the European Heart Rhythm Association diagnostic criteria for AMVP, we did not find an association between different PVC profiles and the presence of severe ventricular arrhythmia (F-HR-PVC 62% vs 57%, I-HR-PVC 38% vs 38%, S-HR-PVC 0% vs 5%, p > 0.05 for all profiles).

### 3.4 Comparison to patients with idiopathic PVCs

For comparison, we included an age- and sex-matched control group of 70 patients with idiopathic PVCs (median age 44 years [IQR 34–56], 76% female). The median PVC burden was 8.0% per 24-hours (IQR 3.0–16.2) (Table 1). In this group, we found F-HR-PVC in 30 patients (43%), I-HR-PVC in 25 (36%) and S-HR-PVC in 15 (21%) (Graphical Abstract, Fig. 4). More patients with MVP had F-HR-PVC (44 patients [63%] vs 30 patients [43%], p = 0.02), and fewer patients had S-HR-PVC (2 patients [3%] vs 15 patients [21%], p = 0.001). The finding of higher maximum heart rate and lower minimum heart rate in the MVP population was not found in the control group.

NSVT was present in 24 (34%) of the idiopathic PVC patients and was present in all three PVC profiles. However, both the F-HR-PVC and the I-HR-PVC had significantly higher rate of NSVT compared to the S-HR-PVC (IRR 9.5 [95% CI 1.2–74.0], p = 0.03, and IRR 63.2 [95% CI 7.7–501.0], p < 0.001, respectively).

## 4 Discussion

This is the first study to show the relationship between heart rate and PVC burden in patient with MVP. F-HR-PVC was the most common PVC profile, while S-HR-PVC was rare, in contrast to the control group of patients with idiopathic PVCs who showed a more uniform distribution of PVC profiles.

Patients with MVP and a F-HR-PVC profile had a higher rate of NSVT, even with a similar PVC burden as I-HR-PVC. In the control group patients with both F-HR-PVC and I-HR-PVC had higher rate of NSVT compared to the S-HR-PVC. Higher PVC burden and higher rate of NSVT were associated with severe ventricular arrhythmia. However, we found no association between the different PVC profiles and severe ventricular arrhythmia. These findings suggest heterogeneity regarding the mechanisms for ventricular arrhythmias in the MVP population.

### 4.1 PVC profiles in MVP patients

F-HR-PVC was the most common profile and was found in 63% of patients. This prevalence is comparable to other studies in unselected patient populations [8], [13], [15]. F-HR-PVC was observed in 50% of unselected patients undergoing Holter monitoring [13], 42% of symptomatic chronic PVC patients [15], and in 79% of patients with chronic PVCs referred for participation in antiarrhythmic drug therapy trials [8]. We found I-HR-PVC in 34% of our patients, consistent with findings from previous studies [13], [15]. However, none of these older studies included a significant number of MVP patients.

The prevalence of S-HR-PVC profile was rare (3%) and less than previous reported prevalence of 20% and 14% [8], [13]. This was more in line with the results from the idiopathic PVC patients, with a prevalence of S-HR-PVC of 21%. These findings may indicate that S-HR-PVC profile is a phenotype less common in MVP patients. MVP patients with S-HR-PVC had no severe ventricular arrhythmia, consistent to previous data on S-HR-PVC [13]. However, whether S-HR-PVC is associated with lower risk in MVP patients remains to be explored.

The methods of establishing baseline heart rate between PVC profiling studies are heterogeneous [8], [13], [15]. One study used the cycle length before individual beats preceding the PVCs, another used 1-minute intervals, while a third used mean hourly heart rate. In this study we applied the latter method of using mean hourly heart rate. This method is less prone to beat-to-beat factors such as acute autonomic changes and compensatory pauses [13], and more applicable in periods of bigeminy and trigeminy, frequently found in patients with MVP.

### 4.2 Arrhythmic mechanisms and PVC profiles

The presence of different relationships between PVC burden and heart rate in MVP patients could suggest different underlying arrhythmic mechanisms. The F-HR-PVC profile could be affected by catecholamine activity, the S-HR-PVC profile could be evoked by vagal activity, whereas the I-HR-PVC could more likely be independent of autonomic activity variations [13]. Interestingly, some studies infer that there might be a functional abnormality of the autonomic nervous system in patients with MVP, or that a neuroendocrine dysfunction could be present [4], [20]. It has been reported a reduced vagal tone during daily activities in the MVP patient, but the autonomic abnormalities are not uniform [21]. Similarly, another study suggested sympathetic overactivity and a decreased vagal tone in MVP [22]. We found a higher maximum heart rate, and a lower minimum heart rate, in the F-HR-PVC profile than in the I-HR-PVC profile. This could infer that the F-HR-PVC profile have abnormalities in the autonomic nervous system, such as a combination of reduced vagal tone and increased catecholamine activity. Interestingly, all patients with NSVTs during exercise ECG had a F-HR-PVC profile, inferring F-HR-PVC as a possible more arrhythmogenic profile and strengthens the theory of a catecholamine-sensitive mechanism acting as trigger for ventricular arrhythmias with this profile.

### 4.3 Comparison of the arrhythmic risk between different PVC profiles

We found no association between different PVC profiles and the presence of severe ventricular arrhythmia in MVP patients. This result was also consistent in the analysis of the AMVP patients. Greater PVC burden and NSVT burden was associated with severe ventricular arrhythmia, similar to previous data from this cohort [7], emphasizing PVC burden and presence of NSVT as risk stratification factors in AMVP.

Our finding of a high prevalence of F-HR-PVC profile among MVP patients could contribute to a better understanding of the mechanisms behind ventricular arrhythmias in this population. Even though we found no association between F-HR-PVC and severe ventricular arrhythmias, the F-HR-PVC profile seemed more arrhythmogenic due to their higher NSVT burden, even with similar PVC burden.

### 4.4 PVC morphology in MVP patients

We found a high prevalence of PVCs with right bundle branch morphology with superior axis suggesting an origin from the papillary muscles or inferolateral left ventricular wall, as previously described [1], [7]. The theoretical arrhythmic mechanism in AMVP includes mechanical stretch of the papillary muscles that may decrease resting diastolic potential leading to stretch-activated early afterdepolarizations resulting in triggered activity [23], [24]. There was similar prevalence of different PVC morphology in the F-HR-PVC and I-HR-PVC profile, which might suggest that the mechanical stretch theory may be independent of autonomic tone and neurohumoral factors. Additionally, the stretch theory also implies a reactive response in the papillary muscles and surrounding myocardium, leading to regionalized fibrotic areas that could act as substrates for re-entry tachycardias independent of autonomic tone [1]. This could explain why we found higher NSVT burden in patients with F-HR-PVC. There was a relatively high frequency of PVCs with suggested origin from the outflow tracts. The relation between PVC origin and AMVP is not fully explored and the impact of PVCs from outside of the mitral valve apparatus in risk stratification for severe ventricular arrythmia in AMVP is unknown.

### 4.5 Clinical implications

Our study showed that most MVP patients undergoing Holter monitoring had F-HR-PVC, and rarely S-HR-PVC. These insights could have implications on our understanding of arrhythmogenesis in AMVP. It remains to be explored whether profiling Holter recordings could be helpful in choosing antiarrhythmic therapy in MVP patients. Our findings may be of value in the planning of catheter ablation procedures in these patients, as well as randomized controlled trials for finding potential effective medical therapy since previous studies indicate that the PVC profiles show different efficacy for betablocker therapy [13], [15].

### 4.6 Limitations

This study had an ambispective observational design with inherent limitations, including recruitment, referral and recall bias. The sample size also limits the robustness and generalizability of the results. Our findings should be validated in larger and independent cohorts of arrhythmic MVP.

We included all patients with ≥100 PVCs per 24-hours, which is lower than the European Heart Rhythm Association diagnostic criteria for AMVP [1]. This threshold was chosen to evaluate the clinical use of PVC profiles even in patients with a low PVC burden, but a high enough PVC burden to be reliably classified (Fig. 4). Our study was not a follow-up study and the modulation of arrhythmias by medication is thus unknown. We do not have information about the amount and timing of physical exercise during the Holter monitoring. Furthermore, the possibility that patients in the I-HR-PVC profile could have been in less physical activity during the Holter monitoring, and thus not profiled as F-HR-PVC, cannot be excluded.

### 4.7 Conclusion

This is the first study to show that fast-HR-dependent-PVC was the most common PVC profile in MVP patients, suggesting a catecholamine-sensitive mechanism acting as trigger for ventricular arrhythmias in these patients. Slow-HR-dependent-PVC profile was rare. Different PVC profiles did not seem to infer higher risk of severe ventricular arrhythmia in our study.

## CRediT authorship contribution statement

**Cecilie Bugge:** Writing – review & editing, Writing – original draft, Visualization, Project administration, Methodology, Investigation, Formal analysis, Data curation, Conceptualization. **Christian K. Five:** Writing – review & editing, Methodology, Investigation, Formal analysis, Data curation, Conceptualization. **Tariq Ahmed:** Writing – review & editing, Investigation, Data curation, Conceptualization. **Petter M.J. Skar:** Writing – review & editing, Investigation, Data curation, Conceptualization. **Anna Isotta Castrini:** Writing – review & editing, Methodology, Investigation, Data curation, Conceptualization. **Margareth Ribe:** Writing – review & editing, Investigation, Data curation, Conceptualization. **Nina E. Hasselberg:** Writing – review & editing, Methodology, Investigation, Data curation, Conceptualization. **Lars A. Dejgaard:** Writing – review & editing, Methodology, Investigation, Data curation, Conceptualization. **Mathis K. Stokke:** Writing – review & editing, Supervision, Resources, Project administration, Methodology, Funding acquisition, Data curation, Conceptualization. **Kristina H. Haugaa:** Writing – review & editing, Supervision, Resources, Project administration, Methodology, Investigation, Funding acquisition, Conceptualization. **Eivind W Aabel:** Writing – review & editing, Writing – original draft, Visualization, Supervision, Project administration, Methodology, Investigation, Formal analysis, Data curation, Conceptualization.

## Funding

This work was supported by the 10.13039/501100005416Research Council of Norway; Precision 10.13039/100018696Health Center for optimized cardiac care (ProCardio) [grant number 309762].

## Declaration of competing interest

The authors declare the following financial interests/personal relationships which may be considered as potential competing interests: E.W.A. has received speaker honoraria from Boehringer-Ingelheim, AstraZeneca and NovoNordisk; and has served on an advisory board for Boehringer-Ingelheim and AstraZeneca. Author reports no conflict of interest due to this. Other authors report no relationships that could be constructed as a conflict of interest.

## Data Availability

The data underlying this article cannot be shared publicly due to the privacy of individuals that participated in the study. The data will be shared on reasonable request to the corresponding author.
