# Supplementary material for: Premature ventricular complexes increase with heart rate in patients with mitral valve prolapse
Source: Int J Cardiol Heart Vasc. 2026 Apr 15;64:101925. doi: 10.1016/j.ijcha.2026.101925 (PMC13099934; doi:10.1016/j.ijcha.2026.101925)
Supplement: Supplementary Data 1 [file mmc1.docx]

**Supplementary Table 1. Clinical characteristics of MVP patients with and without severe ventricular arrhythmias**

|  | **All**  **(n=70)** | **Without severe ventricular arrhythmia**  **(n=57)** | **With severe ventricular arrhythmia**  **(n=13)** | **P-value** |
| --- | --- | --- | --- | --- |
| Age at inclusion, years (IQR) | 48 (35-58) | 48 (35-60) | 40 (30-57) | 0.21 |
| Female, n (%) | 55 (79) | 45 (79) | 10 (77) | 1.00 |
| EHRA AMVP diagnosis, n (%) | 55 (79) | 42 (74) | 13 (100) | 0.06 |
| PVC profile |  |  |  | 1.00 |
| F-HR-PVC | 44 (63) | 36 (63) | 8 (62) | 1.00 |
| I-HR-PVC | 24 (34) | 19 (33) | 5 (38) | 0.73 |
| S-HR-PVC | 2 (3) | 2 (4) | 0 (0) | 1.00 |
| T-wave inversions inferior wall, n (%) | 10 (14) | 5 (9) | 5 (38) | 0.006 |
| QTc duration, ms | 416 ± 31 | 416 ± 31 | 420 ± 31 | 0.69 |
| **Echocardiography** |  |  |  |  |
| Left ventricular ejection fraction (%) | 57 ± 6 | 57 ± 6 | 54 ± 6 | 0.14 |
| Mitral regurgitation, (grade) |  |  |  | 1.00 |
| None, n (%) | 13 (19) | 12 (21) | 1 (8) |  |
| Mild, n (%) | 37 (53) | 30 (53) | 7 (53) |  |
| Moderate, n (%) | 14 (20) | 11 (19) | 3 (23) |  |
| Severe, n (%) | 6 (9) | 4 (7) | 2 (15) |  |
| Inferolateral mitral annular disjunction, n (%) | 62 (88) | 50 (88) | 12 (92) | 1.00 |
| Inferolateral mitral annular disjunction, length in mm (IQR) | 6 (4-8) | 6 (4-8) | 7 (5-9) | 0.20 |
| Bileaflet MVP, n (%) | 40 (57) | 30 (53) | 10 (77) | 0.13 |
| **Cardiac magnetic resonance imaging (n=47)** | |  |  |  |
| Inferolateral mitral annular disjunction, n (%) | 37 (80) | 29 (81) | 7 (78) | 1.00 |
| Inferolateral mitral annular disjunction, length in mm (IQR) | 7 (6-9) | 6 (5-8) | 10 (8-12) | 0.003 |
| Late gadolinium enhancement, n (%) | 17 (37) | 13 (35) | 4 (44) | 0.44 |
| Papillary muscles, n (%) | 11 (24) | 8 (22) | 3 (33) | 0.37 |
| **PVC morphology (n=69)** |  |  |  |  |
| Right bundle branch block, n (%) | 45 (54) | 35 (61) | 10 (83) | 0.19 |
| Superior axis, n (%) | 38 (58) | 28 (52) | 10 (83) | 0.06 |
| Inferior axis, n (%) | 19 (29) | 13 (24) | 6 (50) | 0.09 |
| Left bundle branch block inferior axis, n (%) | 31 (45) | 26 (46) | 5 (42) | 1.00 |

Values are presented as n (%), median (IQR) or mean ± SD. The P-values were calculated by means of Student t-test, one-way ANOVA, Mann–Whitney U test, χ2 test or Fisher exact test as appropriate.

AMVP=arrhythmic mitral valve prolapse, EHRA=European Heart Rhythm Association, F-HR-PVC=Fast-heart-rate-dependent-PVC, I-HR-PVC=Independent-heart-rate-PVC, IQR=interquartile range, MVP=mitral valve prolapse, NSVT=non-sustained ventricular tachycardia, PVC=premature ventricular complexes, QTc=corrected QT interval, S-HR-PVC=Slow-heart-rate-dependent-PVC, VT=ventricular tachycardia.
